# Corticosteroids in palliative care - perspectives of clinicians involved in prescribing: a qualitative study

**DOI:** 10.1186/1472-684X-13-50

**Published:** 2014-11-18

**Authors:** Anne Denton, John Shaw

**Affiliations:** School of Pharmacy, Faculty of Medical and Health Sciences, The University of Auckland, Building 505, 85 Park Road, Auckland, Grafton New Zealand

**Keywords:** Corticosteroids, Palliative care clinicians, Hospices, Prescribing, Qualitative study

## Abstract

**Background:**

Corticosteroids are commonly prescribed in palliative care for alleviation of both specific and non-specific symptoms, but relatively little is known of the perspectives of clinicians and what influences their prescribing in this context. The aim of this study was to explore the experiences and perspectives of those involved in the prescribing of corticosteroids in palliative care.

**Methods:**

Semi-structured interviews were undertaken with 12 medical practitioners and six senior nurses from a sample of six New Zealand hospices to identify their experiences and attitudes regarding the prescribing of corticosteroids. A general inductive approach was used to thematically analyse data.

**Results:**

Five broad themes were identified: the role of corticosteroids in palliative care; indications for corticosteroids; influences on prescribing; use of guidelines; and perceptions of previous study data on prescribing patterns for their hospice. Interviewees regarded these agents as having an important place in in palliative care but expressed a degree of uncertainty about certain aspects of their use. They were concerned about issues such as prescribing for non-specific indications, methods of stopping, and lack of monitoring and reviewing. Guidelines were used routinely by only one of the sample hospices. Corticosteroids tended to be prescribed experientially or by habit, rather than based on evidence-based guidelines.

**Conclusions:**

This study has highlighted differences in the understanding of the place of corticosteroids in palliative care by the clinicians interviewed in this study and different practices, particularly in the treatment of non-specific symptoms and in the use of guidelines. These findings suggest a need for further research and discussion about the role of corticosteroids in palliative care and the development of evidence-based guidelines to assist prescribers.

## Background

Corticosteroids are a potent group of medicines which have been used in palliative care since the late 1950s to alleviate both specific and non-specific debilitating symptoms [1–5]. The proportion of palliative care patients prescribed corticosteroids has been reported as ranging from 32% to 80%, with a median of about 60% [1–4, 6–13]. In a recent New Zealand study of corticosteroid prescribing in palliative care, we observed that 65% of patients received at least one course of corticosteroids and there was a marked consistency in prescribing frequency across the six sample hospices (range: 61-69%) [14]. There were similarities between the hospices in the choice of agents, dose ranges, and indications for use, however, they differed considerably in the use of guidelines, recording of adverse effects, review and monitoring, and the process for stopping these medicines [14].

A number of authors have suggested that prescribing of corticosteroids is unsupported by rigorous evidence and particular concern has been expressed about their ‘non-specific’ use [2, 15–23]. Caution has to be exercised regarding this issue as there are different perceptions of what constitutes ‘non-specific’ , with some authors restricting the definition to anorexia-cachexia symptoms (anorexia, fatigue, weight loss), whereas others include pain, dyspnoea and poor wellbeing in this category [24, 25]. In our previous study, the term non-specific was used when the reasons for prescribing were not clear, that is when there was no record of the cause of the symptom. If, for example, pain was due to spinal cord compression, or dyspnoea due to lymphangitis, then this was recorded as a specific indication for corticosteroids. Using the broader definition, we found that 40.4% of patients in the New Zealand study received corticosteroids for non-specific symptoms, with ‘general wellbeing’ being a frequent indication [14].

The adverse effects of corticosteroids are well-known, as are the challenges associated with choice of agent, dose, duration, route, monitoring and abrupt cessation [1, 3, 8, 12, 13, 20–22, 26–30]. Clinicians must balance the potential benefits and harm associated with these potent drugs and guidelines have been developed to assist decision-making and curb the potential for inappropriate prescribing [8, 31]. Lundstrom and Furst (2006) have argued that guidelines need to be evidence-based to ensure the best outcomes for patients with minimum adverse effects, as well as being easy to follow, clinically relevant, comprehensive, and flexible [2]. In our previous study, guidelines were available at most sites but only one of the six hospices used them routinely [14].

Despite the considerable literature on the benefits and drawbacks of corticosteroids in palliative care, and on how they are used in clinical practice, relatively little is known of what influences prescribers in their decisions to employ these agents. For instance, is prescribing evidence-based or largely based on experience and habit? Are guidelines available for prescribers and do they use them? What influence do non-medical colleagues such as senior nurses have on prescribing decisions?

The aim of this qualitative study was to explore clinicians’ perceptions of the role of corticosteroids in palliative care, and the main influences on their prescribing. This paper represents the second phase of a larger study on corticosteroid prescribing and was designed to complement the first phase, a retrospective analysis of corticosteroid prescribing in a sample of New Zealand hospices, which was reported previously in this journal [14]. A feature of this mixed-methods approach was that participants were shown summary data from the retrospective study pertaining to both their individual hospice and the full sample of hospices, allowing them to reflect on their actual practice in this area.

## Methods

### Research design

The study was approved by the New Zealand Multi-regional Ethics committee (MEC/08/37/EXP). Semi-structured interviews with palliative care clinicians (both doctors and senior nurses) were undertaken to elicit their perspectives on the prescribing of corticosteroids in palliative care. This qualitative approach allowed more detailed exploration of their opinions and experiences than by other methods.

### Setting and participants

The setting was six hospices in the North Island of New Zealand as described in the previous study [14] and 18 experienced clinicians were recruited using a purposive sampling method [32]. Written consent to participate was obtained from each clinician before commencing and the hospice directors had given previous consent for participation of their sites. Three clinicians were recruited from each hospice, comprising two medical practitioners (including the Medical Director) (M1 to M12) and one senior palliative care nurse (N1 to N6). The rationale for including senior nurses was that while they were not prescribers themselves, they did contribute to team decisions on corticosteroid prescribing.

### Data collection

An interview guide (Table 1) was developed based on a review of the literature and, in part, from the findings of the previous study of actual corticosteroid prescribing in the same sample hospices [14]. Before the interview, interviewees were provided with summary data from the previous study which were specific to each hospice, but also compared their hospice to the average of all the sample hospices.

**Table 1:** **Semi-structured interview questions frame**

Section	Questions
1.	Role and background of interviewee
2.	Personal philosophy of corticosteroid prescribing in palliative care
3.	Knowledge and understanding of corticosteroids
4.	Influences on prescribing and choice of corticosteroids in palliative care
5.	Their perceptions of the previous study data for their hospice

The interview guide was pre-tested to ensure that the questions were clear. The interviews were conducted during 2010 by the first author (AD) face-to-face at the participants’ home hospice. Interviews lasted about 45–60 minutes and were audio-recorded and transcribed. After transcription, the written data was checked (by a second researcher) against the digital recordings, then re-checked by the interviewer to address inaccuracies.

### Data analysis

Each transcript was entered on an NVivo 8 qualitative data analysis programme. A general inductive approach was used and broad themes were identified from the initial readings of the transcripts and a basic coding structure was developed by the first author (AD). The codes were reviewed by the second author (JS) and colleagues versed in qualitative research. The authors then aggregated similar narratives and developed themes that represented the beliefs, opinions and understandings of the clinicians. With 18 interviews, there was considerable overlap of data, indicating data saturation. Once the over-arching themes were finalised, they became the content of the qualitative research.

## Results

Seven of the medical practitioners were palliative care specialists, and five were general practitioners (family physicians) who did not have specialist palliative care qualifications but were very experienced. The six nurses were senior palliative care nurses. The six sample hospices included both larger urban and smaller rural locations and ranged in size from five to 18 beds; the sample hospices were considered to be representative of hospices throughout New Zealand (32 in total); these are described in more detail in the previous paper [14]. Five broad themes emerged from the analysis: the role of corticosteroids in palliative care; indications for corticosteroids; influences on prescribing; use of guidelines; and perceptions of previous study data for their hospice.

### Role of corticosteroids in palliative care

There was general agreement that corticosteroids were prescribed differently in palliative care, with a broader range of indications than general medicine:

“I think we have a lower threshold for prescribing steroids than general medicine.” (M11)

Most participants supported short-term prescribing of corticosteroids and a few commented that all palliative care patients should have a course of corticosteroids at some stage of their palliative care:

“Steroids are not the answer. They are the stop-gap until you figure out the answer for a lot of our patients.” (M1)

“It’s almost said that you don’t get a good death without somebody having had a honeymoon of steroids at some stage.” (M12)

Several interviewees commented about familiarity/casualness in long-term usage and observed that it was easy once they had been added to a patient’s regimen for them to be forgotten:

“I think drugs like steroids can slip under the radar a bit.” (M12)

In contrast, others thought that they had become more aware of their potency and were becoming more considered and less liberal in their use:

“I think there was a time we used to think it was a bit of a wonder drug and we just threw it in there, but we actually think a little bit more about what we are trying to achieve nowadays and probably use it for the right reasons.” (N2)

### Indications for corticosteroids

Many suggested they tried to be clear why the corticosteroid was being used and that it would be for a specific indication:

“I try to know the indication I am using when I am prescribing steroids. Try to be clear to myself but also to the team, what I am trying to achieve. What the indication is.” (M6)

All interviewees were confident with prescribing corticosteroids for specific indications such as neurological symptoms or bowel obstruction. In contrast, some dissented when prescribing for non-specific indications was suggested, for instance for ‘general wellbeing’. Most respondents explained that within the label of ‘general wellbeing’ there were some very specific reasons for prescribing:

“We do use them a lot for a sense of wellbeing but looking at appetite as well. That is the reason we prescribe them. To try and improve appetite and with that comes a sense of wellbeing.” (M5)

“Loss of energy, loss of appetite and wellbeing to me is an indication that I could prescribe steroids for.” (M6)

When asked if a corticosteroid was ever prescribed as a ‘comfort drug’ , there were mixed responses. A number of respondents were unhappy with this term and felt that ‘comfort’ came as a result of specific symptom management:

“They are a symptom management drug and I think that is what we are in the business of. If that provides comfort then that is a bonus.” (M7)

Some participants suggested the corticosteroid was often prescribed for the ‘comfort’ of the clinician rather than the patient.

“Perhaps we did see it as a comfort drug, not for the patients but for us, so that we are actively doing something.” (N2)

When asked if a corticosteroid could be described as a ‘fix it all’ drug, most interviewees replied in the negative:

“No. I would never just prescribe them as, oh well I can’t think of anything better let’s try … No - I don’t see them as a ‘fix-it-all’. They are too dangerous to do that with. They have too many side-effects.” (M2)

Most interviewees felt that adverse effects were inevitable and were frequently not identified until they were very obvious, for example Cushingoid syndrome:

“It’s the long term side effects that are actually important and they are subtle and sneak up on you.” (M1)

### Influences on prescribing

When asked if prescribing in palliative care was evidence-based, opinions varied, with the majority unsure of the rigour of the evidence, while a few felt the evidence was strong:

“I don’t know if it is really - we try to be evidence based but it is not very clear cut.” (M9)

“There are quite a few solid articles now about the use of corticosteroids for cancer patients, for symptom management, adjuvant therapy.” (N1)

“I don’t think the evidence is out there actually.” (M2)

When discussing intuitive and anecdotal prescribing of corticosteroids, all but one agreed that these were major factors in prescribing:

“I suspect so. I wonder sometimes, can you always have a scientific reason for everything?” (N6)

Only one specialist stated that their prescribing was specifically based on clinical findings:

“No. I think that the prescribing here would first of all be based on clinical findings ….. It is not really a gut feeling.” (M5)

Prior experience was a major determinant of corticosteroid prescribing, for example, medical practitioners who had worked internationally responded that dexamethasone was the corticosteroid they had used in other countries, and they believed there was no evidence to suggest a change in practice in New Zealand:

“Every environment I have been in they were using dexamethasone … I stick to the devil I know rather than trying to find out about the devil I don’t.” (M11)

Some respondents said prescribing was an ‘institutional habit’ and each unit appeared to have its unique culture:

“We work in a team and we try and get consistency of prescribing. If you try and get medical practitioners to prescribe the same way, that’s like herding cats.” (M11)

### Guidelines

Five hospices reported use of guidelines but these varied considerably in their use and interpretation. One had guidelines that were used routinely, others used them for a few specific indications only, or had guidelines but didn’t use them. Only one hospice chose not to have guidelines as they preferred to prescribe individually for their patients.

“We have protocols, which are agreed upon by the medical team, and then the team. When we initiate steroids, we prescribe them on a tapering protocol to zero. So that is how they get prescribed from onset to completion.” (M6)

“Yes we do have guidelines for different situations. Certainly we have got one for spinal cord compression.” (M7)

“I do not do guidelines particularly well. I’m not saying if it is a good thing or a bad thing. I don’t think we have steroid guidelines.” (M1)

“I am not a fan of hard and fast rules – [such as] protocols for steroids, because you are weighing up so many things and it is not about the physical stuff – often it is about quality as well.” (M2)

### Perceptions of previous study data for their hospice

Interviewees were asked to reflect on the data on actual prescribing for their hospice (described in Methods). Some of their perspectives are listed in Table 2.

**Table 2:** **Interviewees’ perceptions of their individual hospice data**

Data	Responses
The proportion of patients prescribed corticosteroids across the six hospices (range: 61% to 69%)	The interviewees were mostly surprised where their relative corticosteroid usage lay:
	*“Interesting. I would not have put us as great steroid users… we are leading the ranks on this study.” (M1)*
	*“I thought probably the use in our [hospice name] was quite high and it is lower than the other hospices so that surprises me.” (M4)*
	*“I think that is very representative.” (M5)*
	*“I was a little surprised it [the range] was so narrow.” (N3)*
The proportion of corticosteroids prescribed for non-specific/general wellbeing indications (range: 33% to 61%)	All were surprised at the proportion of patients being prescribed corticosteroids for non-specific/general wellbeing indications and some went so far as to say they found it disappointing:
	*“I am surprised that general well being is so high.” (M12)*
	*“45% for wellbeing. Well that is disappointing.” (M3)*
The proportion of corticosteroid adverse effects not recorded. (range: 55% to 85%)	There was no surprise here but it was acknowledged that it was an issue some said they found upsetting:
	*“The ‘not recorded’ it is always an issue.” (M1)*
	*“I was pretty upset with the not recorded.” (M5)*
The method of stopping corticosteroids stopped abruptly - (range: 14% to 34%)	Disappointment was expressed over abrupt cessation of corticosteroids particularly when the patient had been on them for more than three weeks. The general feeling was surprise at the low numbers of patients who had had their corticosteroids reduced gradually:
	*“I was rather taken aback about the abrupt ceasing. That surprised me because that certainly will not be a conscious thing.” (M11)*
	*“I am surprised that gradually is so small.” (M12)*
The proportion of corticosteroids monitored and reviewed (range: 29% to 69%)	Surprise and disappointment were expressed at the low percentage of reviewed and monitored patients:
	*“I was surprised to see that only 57% of the patients were reviewed. I would have thought that it would have been higher than that.” (M4)*
	*“It’s really the ownership of the steroid that’s the issue. We work with oncologists and everybody but the policing and appropriation of the steroid, that is where the system falters.” (M11)*
	*“It doesn’t surprise me that there is a large amount where it has not been recorded. I don’t think that we are very good at recording.” (M2)*

Some interviewees were surprised at where their hospice sat within the range of patients prescribed corticosteroids but were agreeably surprised at its closeness, as this suggested their prescribing was consistent with the ‘norm’. The degree of prescribing for non-specific/general wellbeing indications was commented on with surprise and/or disappointment, although some suggested that when the reason for prescribing was too difficult to explain to a patient, the term general wellbeing was often used.

The small proportion of patients reviewed and monitored was a disappointment to the interviewees who agreed that these were not done well. With the input of a number of teams (e.g. hospice, hospital, Oncology, general practice), they suggested it was not easy for a single team or individual to take an oversight role. There seemed to be a general acceptance that adverse effects were inevitable and therefore not generally recorded in the patient notes; interviewees suggested that the lack of recording was not corticosteroid-specific. They expressed concern at the number of patients who had long-term corticosteroids stopped abruptly and were disappointed with the small number of regimens that were reduced gradually. Most were aware that patients prescribed corticosteroids for more than two to three weeks may have developed adrenal insufficiency.

## Discussion

This qualitative study was set in the wider context of a previous study in which a ‘snapshot’ of actual prescribing was undertaken in the same six hospices where these clinicians were based [14]. A strength of this mixed-methods approach was that interviewees were able to comment on the summary data from the previous study which showed how prescribing practice at their hospice compared to others in the sample. To the best of our knowledge, this is the first qualitative study of its kind, although there have been a number of quantitative studies which have documented corticosteroid prescribing practices [1–5, 7, 9–12]. The interviews with clinicians yielded rich insights into the place of these drugs in palliative care and the main influences on their prescribing. Opinions expressed were diverse and there were few areas in which there was full consensus, perhaps reflecting the nature of the available evidence to guide prescribing.

Interviewees agreed that prescribing of corticosteroids in palliative care was more frequent and more diverse than in general medicine. None were surprised by the relatively high proportions of patients prescribed corticosteroids (about two-thirds) which was in line with reported practice internationally [1–4, 6–13, 33]. Indeed, most took reassurance in this consistency as they presumed it gave credibility to their prescribing. There were mixed opinions on the major influences on their prescribing which were variously described as evidence-based, experiential, anecdotal or intuitive. Many interviewees cited a lack of rigorous evidence and confusing literature and suggested their prescribing of corticosteroids was largely based on the established ‘culture’ at their hospice and particularly influenced by the specialist medical staff.

As previously reported, non-specific symptoms/general wellbeing were the main indications for prescribing in these hospices [14]. These indications (e.g. lack of appetite or fatigue) can difficult to define and, as in the literature, there was some confusion amongst participants as to the meaning of these terms. Some interviewees asserted that there were specific reasons for prescribing under the banner of ‘general wellbeing’ or ‘non-specific’ indications. A recent well-designed trial of dexamethasone has shown significant reductions in cancer-related fatigue in patients with advanced cancer [34]. Given the lack of clarity on what constitutes a non-specific indication, our previous rather negative interpretation of the finding that about 40% of prescribing was for non-specific symptoms, may need to be viewed more positively [14]. Clinicians were firm in their view that the use of corticosteroids as ‘fix it all’ or ‘comfort’ drugs was inappropriate. We believe that further debate on the definitions of ‘non-specific’ , ‘general wellbeing’ and the use of corticosteroids in these contexts is merited.

Most hospices had guidelines for corticosteroid prescribing, but there was considerable variation in their use and interpretation, and only one of the hospices appeared to use them routinely. Whilst guidelines were similar to those used internationally (e.g. at http://www.palliativedrugs.com) (31), interviewees reported that dose reductions, duration, and method of stopping differed between hospices and between prescribers, with little apparent attention paid to guideline suggestions. It is easy to view this as a negative finding; it would, however, be more profitable to undertake further research into the use of guidelines – for example, how useful are they, who uses them, and what are the reasons they are not used more widely?

In our previous study, monitoring and review of corticosteroids was documented in 52% of cases and adverse effects in 32% [14]. When shown this summary data, interviewees were surprised and disappointed at these relatively low levels of recording. They suggested that while review and monitoring were frequently performed, this may not have been recorded in the patients’ notes. Some clinicians conceded that adverse effects were under-recorded and often not identified until a patient was frankly Cushingoid. Only one hospice routinely used a corticosteroid tapering protocol. Poor documentation is not something that is specific to corticosteroid use and is a recurring limitation of all retrospective research. It would be useful to conduct prospective studies of corticosteroid prescribing to determine the true frequency of review and monitoring, and adverse effect recording.

When a patient has been on a corticosteroid for longer than three weeks, the dose should be reduced gradually to avoid an adrenal crisis as abrupt cessation can result in terminal restlessness and anxiety, and even hasten death [20, 28–30]. In our previous study, abrupt cessation of corticosteroids occurred in 23% of all cases and, in approximately half of these, the course was longer than three weeks [14]. Some clinicians were aware that corticosteroids had been abruptly discontinued at a relatively high dose, especially when a patient could no longer swallow, but did not appear particularly concerned with this decision. Although this appears to be acceptable practice internationally, it is both clinically and ethically questionable as the resulting adrenal crisis may increase terminal restlessness [20]. This issue merits further debate, particularly as parenteral formulations of these medicines are available and a route change is possible in these cases [13].

This study has several limitations. It was designed to shed light on how palliative care clinicians (medical practitioners and senior nurses) view the place of corticosteroids in their hospices, and the various factors that influence their prescribing. The clinicians were drawn from a small sample of hospices in New Zealand; these may not be representative of practice in other countries and this limits the generalizability of the findings. In addition, the palliative care community in New Zealand is fairly small and close-knit, which may have led to a degree of uniformity in clinicians’ perceptions and responses. Despite these limitations, some challenging issues in the prescribing of this important group of medicines have been identified and should be further debated in an international context. We suggest that there is a particular need for debate on the use of corticosteroids for non-specific indications and further research on the utility of evidence-based guidelines.

## Conclusion

By exploring New Zealand clinicians’ perspectives of corticosteroid prescribing in palliative care, it was found that guidelines were used infrequently and these medicines tended to be used experientially and anecdotally, rather than based on rigorous evidence. Individual clinicians differed in their corticosteroid prescribing practices on a number of issues, for example dosing, duration and method of stopping. There was some uncertainty about their role in palliative care, particularly for the treatment of non-specific symptoms. These findings suggest that there is a need for further research, particularly around the non-specific prescribing of corticosteroids and the use of evidence-based guidelines to assist prescribers.
